# Rising Above Lumbar Scars: Thoracic Spinal Anesthesia With Hypobaric Bupivacaine for Hip Replacement

**DOI:** 10.7759/cureus.94242

**Published:** 2025-10-09

**Authors:** Richa Chandra, Kartik Sonawane

**Affiliations:** 1 Anaesthesiology, Rohailkhand Medical College and Hospital, Bareilly, IND; 2 Anesthesiology, Ganga Medical Centre and Hospitals, Pvt. Ltd, Coimbatore, IND

**Keywords:** case report, hip replacement, hypobaric bupivacaine, lumbar instrumentation, regional anesthesia, thoracic spinal anesthesia

## Abstract

Neuraxial anesthesia is widely practiced for lower limb, abdominal, and pelvic surgeries due to its favorable risk-benefit profile compared with general anesthesia (GA). However, patients with previous lumbar spine surgery and instrumentation present significant challenges for conventional lumbar spinal anesthesia. Altered anatomy, epidural scarring, and the presence of metallic implants often preclude successful needle placement or result in patchy anesthesia. In such patients, GA may be considered, but it carries substantial risks in elderly individuals with significant comorbidities.

Thoracic spinal anesthesia (TSA), once regarded with apprehension, has recently re-emerged as a feasible and safe technique, particularly when hypobaric local anesthetics are used to direct drug spread toward the lumbar and pelvic dermatomes. We present two geriatric patients with prior lumbar spine surgeries and multiple comorbidities who underwent total hip replacement under TSA with hypobaric bupivacaine and fentanyl. Both patients had uneventful intraoperative courses and smooth postoperative recovery. These cases highlight the potential of TSA with hypobaric solutions as an effective alternative when conventional neuraxial approaches are not possible and GA poses significant risks.

## Introduction

Neuraxial anesthesia remains a cornerstone in anesthetic practice due to its well-documented advantages over general anesthesia (GA). Avoiding airway manipulation reduces the risks of difficult intubation, aspiration, and postoperative respiratory complications [1]. Furthermore, it maintains spontaneous respiration, blunts sympathetic stress responses, and facilitates earlier oral intake, ambulation, and discharge [1]. These benefits are particularly valuable in elderly and frail patients undergoing orthopedic procedures such as hip replacement [2].

Despite these advantages, the administration of spinal anesthesia becomes increasingly complex in patients who have previously undergone lumbar spine surgery with instrumentation. The presence of scar tissue, distorted landmarks, obliteration of epidural spaces, and metallic implants such as pedicle screws and rods complicate needle placement and drug spread. Even if successful needle placement is achieved, the block may remain inadequate due to intradural and extradural adhesions [3].

Traditional alternatives, including lumbar plexus or combined peripheral nerve blocks, may not provide reliable surgical anesthesia for major hip procedures [4]. GA, although technically straightforward, carries a high perioperative risk in elderly patients with multiple comorbidities such as coronary artery disease (CAD), chronic obstructive pulmonary disease (COPD), and diabetes mellitus (DM). Hemodynamic instability, myocardial depression, postoperative delirium, and delayed mobilization often complicate recovery in this vulnerable group [5].

Historically, thoracic spinal anesthesia (TSA) was approached with apprehension because of concerns about potential spinal cord trauma. However, modern anatomical and magnetic resonance imaging (MRI) studies have demonstrated that the posterior subarachnoid space is wider than previously believed at mid-to-lower thoracic levels, particularly around T5-T12, thereby establishing a safe margin for needle placement when performed with fine-gauge needles and meticulous technique [6]. This evolving anatomical understanding has rekindled interest in TSA as a feasible, safe, and segmental alternative in select clinical scenarios.

In this context, TSA with hypobaric bupivacaine emerges as an attractive and innovative option for patients with prior lumbar instrumentation. By exploiting gravitational spread, hypobaric solutions allow comfortable patient positioning with the operative limb upward, provide selective blockade, and minimize hemodynamic compromise [6, 7]. While TSA has been described in various abdominal and breast surgeries [8, 9], its application at the T11-T12 interspace using a low-dose hypobaric bupivacaine-fentanyl combination with an epidural catheter backup for total hip replacement (THR) in patients with altered lumbar anatomy represents a novel approach.

This case report presents two geriatric patients with prior lumbar spine surgery and multiple comorbidities who successfully underwent THR under hypobaric TSA at T11-T12. It highlights both the safety and the clinical feasibility of this technique in circumstances where conventional neuraxial routes are inaccessible and GA poses a significant risk. Through this report, we aim to expand the clinical applicability of TSA using hypobaric bupivacaine and fentanyl, reinforcing its role as a safe, hemodynamically stable, and patient-centered alternative for high-risk surgical populations.

## Case presentation

Case 1

An 80-year-old female presented with a displaced right hip fracture following a fall and was scheduled for THR. She had undergone multiple previous operations on the same hip, including proximal femoral nail fixation twice, followed by removal, and lumbar spine surgery with pedicle-screw instrumentation still in situ. Her comorbidities included CAD with an ejection fraction of 35%, type 2 DM, and hypertension. She appeared frail and undernourished but had no neurological deficits. Airway examination revealed Mallampati class II. Given her poor cardiac reserve and complex spinal history, GA was deemed high risk, while lumbar spinal anesthesia was technically unfeasible.

Case 2

A 78-year-old male presented with a left-sided hip fracture requiring replacement. He had a history of lumbar discectomy, leaving a healed midline scar, but no neurological deficits. His comorbidities included COPD, type 2 DM, and hypertension. He was a former chronic smoker. Upon examination, he was of moderate build, with a Mallampati class II airway. Considering his medical comorbidities and altered lumbar anatomy, a TSA technique was chosen.

Baseline hematological and biochemical profiles of both patients, including mild anemia and borderline renal function with otherwise acceptable perioperative values, are summarized in Table 1.

**Table 1:** Laboratory investigations of two geriatric patients with prior lumbar spine surgery undergoing thoracic spinal anesthesia. Comparative hematological and biochemical parameters of both patients are shown with corresponding reference ranges. Both patients demonstrated mild anemia and elevated serum creatinine, consistent with age-related comorbidities, but were otherwise within acceptable perioperative limits. RBC: red blood cell count; PCV: packed cell volume; MCV: mean corpuscular volume; MCH: mean corpuscular hemoglobin; MCHC: mean corpuscular hemoglobin concentration; PLT: platelet count; PT: prothrombin time; INR: international normalized ratio; SGOT (AST): serum glutamic oxaloacetic transaminase/aspartate aminotransferase; SGPT (ALT): serum glutamic pyruvic transaminase/alanine aminotransferase

Parameter	Reference Range	Patient 1 (80Y/F)	Patient 2 (78Y/M)
Hemoglobin	11.5 – 15.0 g/dl	10.2	9.8
Total Leukocyte Count	4,000 – 11,000/cu.mm	5300	6800
Polymorphs (%)	40 – 75	72	71
Lymphocytes (%)	20 – 45	25	25
Eosinophils (%)	1 – 6	3	4
Monocytes (%)	1 – 8	0	0
Basophils (%)	0 – 1	0	0
Total RBC Count	4.5 – 6.5 million/cu.mm	3.8	3.5
PCV (HCT)	36 – 46 %	32.4	31.2
MCV	80 – 99 fL	83.1	81.9
MCH	26 – 32 pg	26.3	26.1
MCHC	32 – 36 g/dl	31.7	31.2
Platelet Count (PLT)	1.5 – 4.0 lakh/cu.mm	2.17	1.93
Prothrombin Time (PT)	10 – 14 sec	13	12
Control PT	—	14	14
INR	—	0.92	0.85
Serum Creatinine	0.4 – 1.4 mg/dl	1.8	1.6
SGOT (AST)	8 – 40 IU/L	26.5	31.2
SGPT (ALT)	5 – 40 IU/L	31.1	43.7
Plasma Glucose (Random)	70 – 140 mg/dl	162.4	172.9

Procedural overview

**Figure 1:**
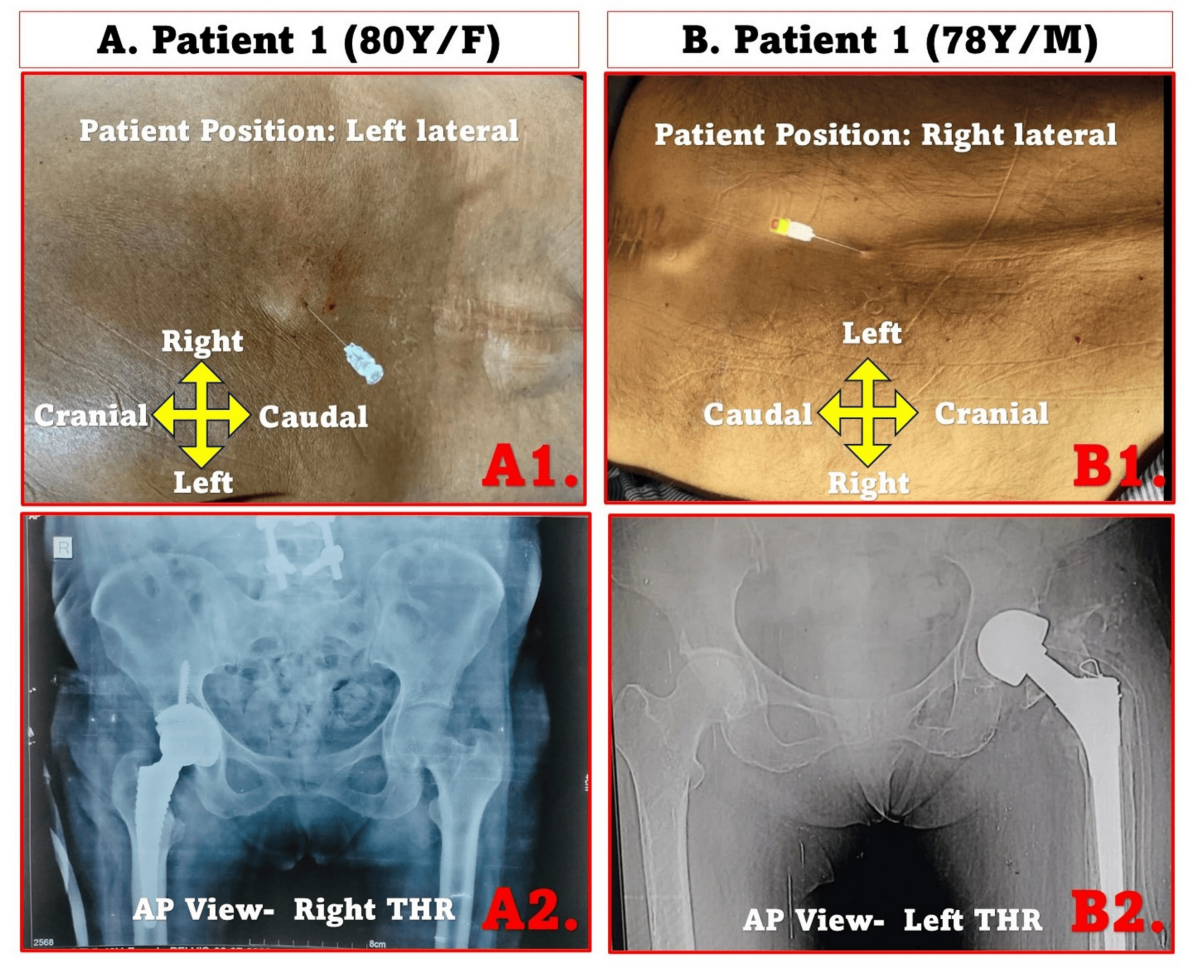
Clinical and radiological findings of two geriatric patients undergoing thoracic spinal anesthesia for total hip replacement. (A1) Spinal puncture performed above the lumbar scar in an 80-year-old female, left lateral position. (A2) Anteroposterior (AP) pelvic radiograph showing right total hip replacement (THR). (B1) Spinal puncture in a 78-year-old male, right lateral position. (B2) AP pelvic radiograph showing left THR. AP: anteroposterior; THR: total hip replacement.

All procedures were performed by a single anesthesiologist (the first author, RC), who has extensive experience in TSA. Both patients were placed in the lateral decubitus position with the operative side up. The clinical and radiological images are shown in Figure 1. Operative records and clinical photographs reveal a healed midline lumbar surgical scar in both patients. In Patient 1, the outline of pedicle-screw instrumentation is clearly visible beneath the scar (Figure 1A1), which is also corroborated by the postoperative radiograph showing spinal implants (Figure 1A2). Patient 2 had previously undergone a non-instrumented discectomy, with a visible midline scar but no spinal hardware on the radiograph (Figure 1B1-1B2). The interspace selected for TSA corresponded to the first clearly palpable, non-scarred thoracic level above the surgical site (T11-T12), chosen to avoid fibrosis or hardware interference while ensuring segmental coverage appropriate for hip arthroplasty (approximately T12-S4).

Thoracic spinal puncture was performed at the T11-T12 interspace, above the lumbar scar, using a 25-gauge Quincke spinal needle (Becton Dickinson, USA) under full aseptic precautions (Figure 1A1-B1). The interspace was identified using surface anatomical landmarks, including the twelfth rib and spinous processes. Neither ultrasound nor fluoroscopic assistance was employed, as the surface anatomy was well defined and preoperative imaging had already delineated the surgical hardware and scar extent. Free flow of clear CSF was obtained before injection, confirming correct intrathecal placement. A total of 1.2 mL of 0.5% isobaric bupivacaine was mixed with 25 µg fentanyl immediately before administration to prepare the injectate. The solution was injected slowly over 10-15 seconds after confirming continuous CSF flow. An epidural catheter was introduced at the same level for potential supplementation and postoperative analgesia.

Patients were kept in the lateral decubitus position with a slight head-down tilt to achieve adequate segmental spread and administered supplemental oxygen (4-6 L/min). Sensory blockade was assessed bilaterally using cold and pinprick discrimination at two-minute intervals. Adequate surgical anesthesia (T8-L2 dermatomes) was achieved within six minutes of injection. Motor blockade was graded using the modified Bromage scale, achieving Grade 2 on the operative limb by 12 minutes. The block remained stable throughout the 90-minute procedure and regressed fully within two hours. Intravenous fentanyl (50 µg) and midazolam (1 mg) were given for anxiolysis. Transient hypotension (MAP 68-72 mmHg) occurred in both patients, corrected promptly with a single 6 mg bolus of mephentermine. Heart rate remained 60-85 bpm, and oxygen saturation >98%. Neither patient experienced bradycardia, desaturation, respiratory discomfort, or postoperative nausea and vomiting. Arterial blood gas (ABG) values were within normal limits (PaO₂ 110-125 mmHg, PaCO₂ 36-38 mmHg). Both patients underwent non-cemented THR (Figure 1 A2, B2), with surgeries completed within 90 minutes. Intraoperatively, each received approximately 400 ml of plasmalyte solution and one unit of packed red blood cells.

Postoperatively, they were managed in a high-dependency unit, with epidural catheters providing effective analgesia. Neurological examinations were performed at 2-, 6-, and 24-hours post-procedure, confirming complete motor and sensory recovery. Neither patient experienced post-dural puncture headache (PDPH), urinary retention, or new neurological deficits. Oral liquids were tolerated after 2 hours, followed by a soft diet at 6 hours. Early mobilization was initiated per protocol, and both patients were discharged on postoperative day 4. Both patients were followed up daily until discharge and reassessed one month later. No delayed neurological symptoms, hemodynamic instability, or wound-related complications were noted, confirming the safety and stability of the thoracic spinal approach in this subgroup.

The perioperative details of both cases are summarized in Table 2. Both patients and their relatives were thoroughly counseled regarding the anesthetic technique, its potential benefits and risks, and informed consent was obtained for the procedure and for publication, while ensuring anonymity.

**Table 2:** Clinical details of two patients undergoing hip replacement after lumbar spine surgery. CAD: coronary artery disease; COPD: chronic obstructive pulmonary disease; DM: diabetes mellitus; EF: ejection fraction; HTN: hypertension; THR: total hip replacement

Parameter	Case 1: 80-year-old Female	Case 2: 78-year-old Male
History of spinal surgery	Lumbar fracture fixation with pedicle screws in situ	Lumbar discectomy, scar present
Orthopedic condition	Right hip fracture, scheduled for THR	Left hip fracture, scheduled for THR
Comorbidities	CAD (EF 35%), DM, HTN	COPD, DM, HTN
Airway	Mallampati II	Mallampati II
Regional block adjunct	Fascia iliaca block (operative side)	Fascia iliaca block (operative side)
TSA level	T11–T12	T11–T12
Intrathecal drug	0.5% isobaric bupivacaine (1.2 ml) + fentanyl 25 μg (hypobaric)	Same
Position	Lateral decubitus, operative side up, slight head-down tilt	Same
Epidural catheter	Yes (for rescue/prolonged analgesia)	Yes
Intraoperative events	Transient hypotension, managed with mephentermine 6 mg	Transient hypotension, managed with mephentermine 6 mg
Surgery duration	~90 minutes	~90 minutes
Postoperative course	Liquids at 2 h, soft diet at 6 h, early mobilization	Similar, uneventful recovery

## Discussion

Patients with previous lumbar spine surgery present one of the most challenging scenarios for anesthesiologists planning neuraxial anesthesia. While spinal and epidural techniques remain the cornerstone of regional anesthesia (RA), their success depends heavily on the presence of normal anatomical landmarks and intact epidural or subarachnoid spaces. Alterations caused by scarring, adhesions, and instrumentation frequently render conventional lumbar approaches unreliable or even impossible. In such circumstances, alternative regional strategies must be considered, with TSA emerging as a promising option.

Challenges of neuraxial anesthesia after lumbar surgery

Spinal and epidural anesthesia are well-established, safe, effective, and operator-friendly approaches that require relatively simple equipment compared to GA. However, in patients with prior lumbar spine surgery, the administration of neuraxial anesthesia becomes complex and sometimes controversial. Scar tissue from previous laminectomies or fusions may distort spinal anatomy, obliterate tissue planes, and hinder needle advancement. It affects both extradural and intradural areas. Extradural adhesions can restrict needle passage or deflect the trajectory, while intradural adhesions alter CSF dynamics and the spread of local anesthetics (LA). Together, these changes increase the risk of technical difficulty, failed insertion, and patchy or incomplete blocks [3]. Sun (1994) emphasized the risk of incomplete sensory blockade in such patients [3]. Metallic instrumentation, such as pedicle screws and rods, adds further complexity by obscuring anatomical landmarks, blocking access to the subarachnoid space, and potentially increasing the theoretical risks of infection spread [3]. Zhu et al. reported the use of ultrasound to facilitate neuraxial access in parturients with prior spine surgery, but this approach requires available unscarred interspaces, which were absent in our patients [10]. An alternative in such patients is to perform GA. However, this option carries significant drawbacks in elderly patients with major comorbidities.

Limitations of general anesthesia (GA) in high-risk patients

**Figure 2:**
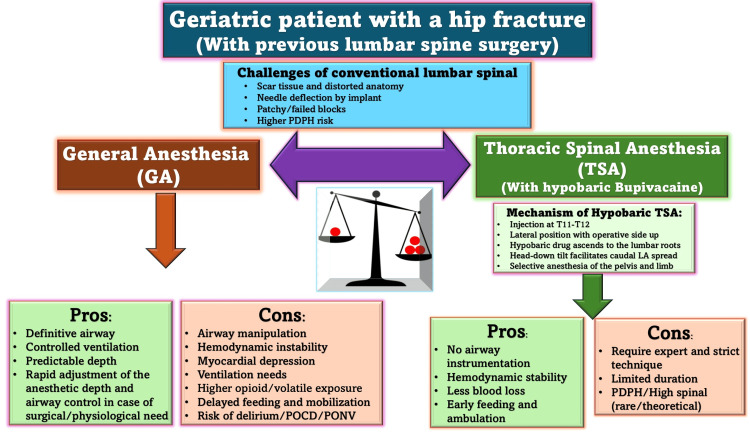
Clinical decision-making in a geriatric patient with a hip fracture and prior lumbar spine surgery. A schematic comparison between general anesthesia (GA) and thoracic spinal anesthesia (TSA) using hypobaric bupivacaine. The diagram highlights the challenges of conventional lumbar spinal approaches in previously operated spines and summarizes the relative advantages and limitations of both techniques. While GA offers airway control and predictable depth, it carries relatively higher risks of hemodynamic instability and delayed recovery in frail patients. TSA, performed at the T11–T12 level using a hypobaric solution, provides segmental anesthesia with better hemodynamic stability and early recovery but requires expertise and has a limited duration. GA: general anesthesia; TSA: thoracic spinal anesthesia; LA: local anesthetic; PDPH: post-dural puncture headache; POCD: postoperative cognitive dysfunction; PONV: postoperative nausea and vomiting. Source: This figure was created by the second author, KS.

GA remains an option when neuraxial techniques fail, but in elderly patients with significant comorbidities, it carries notable risks. Our first patient had ischemic cardiomyopathy with a left ventricular ejection fraction of 35%, while the second had COPD. Both were at risk of perioperative complications such as myocardial depression, arrhythmias, pulmonary compromise, prolonged ventilation, and delayed mobilization. In this context, avoiding airway instrumentation and reducing systemic anesthetic exposure becomes a critical advantage of RA. Radiological evidence of hip prostheses and prior lumbar instrumentation complicated conventional neuraxial access, leading to the choice of TSA. As illustrated in Figure 2, anesthetic management in geriatric patients with prior lumbar spine surgery requires careful balancing between the risks of GA and the advantages of TSA with hypobaric bupivacaine. However, both GA and TSA can be appropriate, depending on anatomical feasibility and the patient’s profile.

Re-emergence of thoracic spinal anesthesia (TSA)

TSA was first introduced by Jonnesco in 1909 but was abandoned for decades due to fears of cord trauma [11]. Modern imaging studies have challenged these concerns. Lee and colleagues demonstrated, through MRI, that the posterior subarachnoid space is wider than previously assumed, with favorable margins at T5 and T10 [12]. Similar studies in Indian populations confirmed these as safe thoracic levels [13]. These findings challenge the dogma of prohibitive risk and reestablish TSA as a feasible option when performed with fine needles and meticulous technique.

Clinical experiences further reinforced the safety and feasibility of TSA. Chandra et al. (2023) reported excellent outcomes in more than 2100 laparoscopic cholecystectomies performed under TSA, while Vincenzi et al. (2023) successfully employed hypobaric TSA for breast surgery [14-15]. Together, these data support TSA as a viable option when conventional lumbar routes are unavailable.

Baricity and its clinical implications

The LA baricity plays a decisive role in TSA spread. Hyperbaric solutions, being denser than CSF, descend under gravity, producing dense blocks on the dependent side [7]. While effective for unilateral surgery, this technique requires positioning the operative side down, a maneuver that is painful and often impractical in patients with hip fractures. Moreover, hyperbaric anesthesia may extend more cephalad, leading to excessive sympathetic blockade and hypotension in frail patients.

Hypobaric solutions, being lighter than CSF, ascend toward the non-dependent side, allowing patients to lie with the injured limb upward- a more comfortable and less distressing position [6]. This approach provides selective blockade of the operative limb, minimizes sympathetic spread, and thereby reduces hypotension [16]. Imbelloni (2014) highlighted that hypobaric agents achieve selective hemianesthesia with fewer hemodynamic consequences [17]. Paliwal et al. described hypobaric spinal anesthesia as “target-specific,” reporting excellent stability in lower limb fractures [18]. Errando et al. (2014) validated its use in elderly patients with hip fractures, showing dose-dependent efficacy while maintaining safety [16]. The key differences between hyperbaric and hypobaric bupivacaine, particularly in the context of TSA for hip surgery, are summarized in Table 3.

**Table 3:** Comparison Between Hyperbaric and Hypobaric Bupivacaine in Spinal Anesthesia. CSF: cerebrospinal fluid; LA: local anesthetic

Feature	Hyperbaric Bupivacaine (Conventional)	Hypobaric Bupivacaine
Baricity	>1.0000 (heavier than CSF)	<1.0000 (lighter than CSF)
Spread direction	Moves downward (dependent side)	Moves upward (non-dependent side)
Patient position for unilateral block	Operative side down (often painful in hip fracture)	Operative side up (comfortable positioning)
Predictability of spread	More predictable, dense motor/sensory block	Slightly less predictable, requires careful positioning
Hemodynamic impact	More sympathetic blockade, higher risk of hypotension	Less sympathetic blockade, greater stability
Block selectivity	Dense bilateral block if the patient supine	More selective unilateral block possible
Duration of block	Often longer, may delay mobilization	Typically shorter, allows earlier recovery
Clinical limitations	Painful positioning, risk of hypotension	Requires preparation of hypobaric solution

Commercial hypobaric formulations are seldom available; therefore, clinicians often rely on practical modifications to achieve relative hypobaricity. This can be accomplished by diluting isobaric bupivacaine with low-density additives such as fentanyl or sterile water rather than warming the solution. Fentanyl, with a baricity of approximately 0.9996 relative to CSF, effectively lowers the overall density of bupivacaine, producing a mildly hypobaric mixture [6]. This simple adjustment has been described in the literature as a reproducible and safe method for achieving selective cephalad spread in lateral decubitus positioning, without the need for specialized equipment or commercial formulations. This method was applied to our patients, offering a simple yet effective solution that did not require specialized equipment.

Alternatives and limitations of other regional techniques

RA for hip surgery can be achieved through lumbar spinal, epidural, combined spinal-epidural, or deep plexus blocks. However, lumbar neuraxial routes may become unreliable or unsafe after spine surgery, and plexus blocks, though valuable, are technically demanding and not always effective as sole anesthetic techniques. Lumbar plexus blocks and related peripheral techniques have been proposed for hip surgery in patients with difficult spines. However, these blocks are technically demanding, lie deep within the psoas compartment, and carry risks of vascular or renal injury [19]. Even under ultrasound guidance, visualization is challenging, and success rates vary widely. Samra et al. (2024) observed that novice anesthesiologists achieved lower success rates with plexus blocks compared to epidural approaches in trauma settings [20]. For major hip replacement procedures, these limitations make plexus blocks less reliable as sole anesthetic techniques. TSA provides a segmental subarachnoid block above the altered lumbar region, avoiding airway manipulation and bypassing scarred spaces. Importantly, our intent is not to claim superiority of TSA over GA or other RA modalities but to highlight its practicality in selected cases where lumbar access is not feasible and general anesthesia carries increased systemic risk.

Clinical outcomes in our patients

In both cases, hypobaric TSA provided excellent surgical conditions, minimal hemodynamic compromise, and a smooth recovery. Only transient hypotension was observed, easily corrected with small doses of mephentermine. The addition of an epidural catheter provided flexibility for rescue anesthesia and effective postoperative analgesia. Both patients tolerated early oral intake, resumed diet within six hours, and mobilized early with physiotherapy. These outcomes demonstrate the tangible benefits of TSA in enhancing recovery and reducing morbidity.

Choice of interspace (T11-T12)

The puncture level at T11-T12 was selected as the first clearly palpable and non-scarred thoracic interspace above the lumbar surgical scar, thereby avoiding dense fibrotic planes or instrumentation that could compromise safety or needle trajectory. This level also ensures segmental coverage of the T12-S4 dermatomes, encompassing the iliohypogastric, femoral, obturator, and sciatic components required for THR. By administering a mildly hypobaric intrathecal mixture in the lateral decubitus position, cephalad spread to T12-L1 and caudal migration to the sacral roots were achieved, providing selective anesthesia of the operative limb with limited sympathetic involvement.

Broader implications

The growing body of literature, including contributions by Chandra, Vincenzi, Imbelloni, Errando, and Paliwal, supports the broader use of TSA with hypobaric solutions in selected patients. Concerns about cord injury remain valid but can be mitigated through careful patient selection, the use of fine-gauge needles, strict adherence to positioning principles, and judicious dosing.

Beyond its immediate application in patients with altered lumbar anatomy, TSA holds promise for several high-risk surgical populations. Studies have demonstrated its feasibility in laparoscopic cholecystectomy, breast surgery, and urological and lower abdominal procedures. These findings suggest that, when performed with appropriate dosing and vigilant monitoring, TSA can achieve segmental, stable, and targeted anesthesia while minimizing airway manipulation and hemodynamic fluctuations. Its use in geriatric and cardiac-compromised patients, where GA carries disproportionate risks, represents an expanding frontier in RA.

In routine practice, hyperbaric bupivacaine remains the most widely used due to its predictability and availability. However, in geriatric patients with hip fractures and altered lumbar anatomy, hypobaric TSA provides a position-friendly, hemodynamically stable, and target-specific alternative that can expand the boundaries of RA.

Advantages and limitations

Our cases highlight several advantages of TSA with hypobaric bupivacaine. The technique allowed comfortable positioning with the operative side up, avoiding the pain associated with dependent positioning required for hyperbaric anesthesia. Hemodynamic stability was maintained, with only transient hypotension requiring minimal vasopressor support. The addition of an epidural catheter provided flexibility for rescue anesthesia and effective postoperative analgesia. These features, combined with rapid recovery and early mobilization, underscore the clinical utility of hypobaric TSA in elderly patients with altered lumbar anatomy.

However, important limitations must be acknowledged. This report describes only two cases, limiting the generalizability of the findings. Hypobaric formulations are not commercially available and require manual preparation, which may introduce variability. Although no neurological or respiratory complications were observed, TSA carries theoretical risks of cord trauma and high spinal block if performed without meticulous technique. Finally, although the outcomes were favorable in our patients, larger prospective studies are needed to establish standardized dosing regimens, assess long-term safety, and determine the broader applicability of this technique.

## Conclusions

These cases illustrate that TSA using a mildly hypobaric mixture of bupivacaine and fentanyl can be a safe and feasible anesthetic option in carefully selected patients with previous lumbar spine surgery, where conventional neuraxial access is technically unfeasible and GA poses an elevated risk. The success of this technique depends on experienced practitioners, meticulous patient positioning, and adherence to strict procedural safety principles. While both cases demonstrated favorable outcomes without neurological or hemodynamic complications, these findings should be interpreted as exploratory observations rather than definitive proof of safety or efficacy. Larger prospective studies with detailed procedural and follow-up data are warranted to establish standardized protocols and confirm long-term safety.

## References

[1] Olawin AM, Das JM (2025). Spinal Anesthesia. [Updated 2022 Jun 27]. https://www.ncbi.nlm.nih.gov/books/NBK537299/.

[2] Cheung KY, Yang TX, Chong DY, So EH (2023). Neuraxial versus general anesthesia in elderly patients undergoing hip fracture surgery and the incidence of postoperative delirium: A systematic review and stratified meta-analysis. BMC Anesthesiol.

[3] Sun KO (1994). Spinal anaesthesia following previous spinal surgery. Eur J Anaesthesiol.

[4] Polania Gutierrez JJ, Ben-David B (2025). Lumbar Plexus Block. [Updated 2023 Jan 29]. https://www.ncbi.nlm.nih.gov/books/NBK556116/.

[5] Yadav S, Jahagirdar A, Jamwal P, Mishra J, Thind GB, Shashank C, Tiwari R (2024). Retrospective study of anesthesia-related complications in elderly patients undergoing surgery. J Pharm Bioallied Sci.

[6] Imbelloni LE, Gouveia MA (2014). A comparison of thoracic spinal anesthesia with low-dose isobaric and low-dose hyperbaric bupivacaine for orthopedic surgery: A randomized controlled trial. Anesth Essays Res.

[7] Loubert C, Hallworth S, Fernando R, Columb M, Patel N, Sarang K, Sodhi V (2011). Does the baricity of bupivacaine influence intrathecal spread in the prolonged sitting position before elective cesarean delivery? A prospective randomized controlled study. Anesth Analg.

[8] Ellakany MH (2014). Thoracic spinal anesthesia is safe for patients undergoing abdominal cancer surgery. Anesth Essays Res.

[9] Paliwal N, Maurya N, Suthar OP, Janweja S (2022). Segmental thoracic spinal anesthesia versus general anesthesia for breast cancer surgery: A prospective randomized-controlled open-label trial. J Anaesthesiol Clin Pharmacol.

[10] Zhu G, Wang X, Yang L (2023). Real-time ultrasound-guided neuraxial anesthesia for cesarean section in parturients with previous internal fixation surgery for lumbar fracture: A case series. Quant Imaging Med Surg.

[11] Imbelloni LE (2010). JONNESCO: One century of thoracic spinal anesthesia history. Rev Bras Anestesiol.

[12] Lee RA, van Zundert AA, Breedveld P, Wondergem JH, Peek D, Wieringa PA (2007). The anatomy of the thoracic spinal canal investigated with magnetic resonance imaging (MRI). Acta Anaesthesiol Belg.

[13] Singh R, Srivastva SK, Prasath CS, Rohilla RK, Siwach R, Magu NK (2011). Morphometric measurements of cadaveric thoracic spine in Indian population and its clinical applications. Asian Spine J.

[14] Chandra R, Misra G, Datta G (2023). Thoracic spinal anesthesia for laparoscopic cholecystectomy: An observational feasibility study. Cureus.

[15] Vincenzi P, Stronati M, Garelli P, Gaudenzi D, Boccoli G, Starnari R (2023). Segmental thoracic spinal anesthesia for laparoscopic cholecystectomy with the "Hypobaric" technique: A case series. Local Reg Anesth.

[16] Errando CL, Soriano-Bru JL, Peiró CM, Ubeda J (2014). Single shot spinal anaesthesia with hypobaric bupivacaine for hip fracture repair surgery in the elderly. Randomized, double blinded comparison of 3.75 mg vs. 7.5 mg. Rev Esp Anestesiol Reanim.

[17] Imbelloni LE (2014). Spinal hemianesthesia: Unilateral and posterior. Anesth Essays Res.

[18] Paliwal N, Kokate MV, Deshpande NA, Khan IA (2024). Spinal anaesthesia using hypobaric drugs: A review of current evidence. Cureus.

[19] Gadsden JC, Lindenmuth DM, Hadzic A, Xu D, Somasundarum L, Flisinski KA (2008). Lumbar plexus block using high-pressure injection leads to contralateral and epidural spread. Anesthesiology.

[20] Samra T, Aditya A, Amar PK, Jain K, Saini V, Naik B N (2024). Ultrasound-guided lumbar plexus-sciatic nerve blocks versus epidurals for orthopaedic surgeries: A study to compare the competency of novice anaesthesiology residents in a high-volume level 1 trauma centre. Cureus.

